# Linear-Time Direct Data Assignment Algorithm for Passive Sensor Measurements

**DOI:** 10.3390/s19245347

**Published:** 2019-12-04

**Authors:** Chaoxin He, Min Zhang, Guizhou Wu, Fucheng Guo

**Affiliations:** State Key Laboratory of Complex Electromagnetic Environment Effects on Electronics and Information System, National University of Defense Technology, Changsha 410073, China; hechaoxin12@nudt.edu.cn (C.H.); wuguizhouacademic@163.com (G.W.); gfcly@21cn.com (F.G.)

**Keywords:** data association, multi-sensor, multi-target tracking, passive sensor

## Abstract

To solve the problem of passive sensor data association in multi-sensor multi-target tracking, a novel linear-time direct data assignment (DDA) algorithm is proposed in this paper. Different from existing methods which solve the data association problem in the measurement domain, the proposed algorithm solves the problem directly in the target state domain. The number and state of candidate targets are preset in the region of interest, which can avoid the problem of combinational explosion. The time complexity of the proposed algorithm is linear with the number of sensors and targets while that of the existing algorithms are exponential. Computer simulations show that the proposed algorithm can achieve almost the same association accuracy as the existing algorithms, but the time consumption can be significantly reduced.

## 1. Introduction

The problem of passive sensor data association, that is, deciding which measurement derived from which target in a multi-sensor multi-target tracking problem has been investigated for many years in radar, reconnaissance and wireless communications [1,2,3,4,5], etc. The objective of multi-sensor multi-target tracking is to detect an unknown number of targets and estimate their states using measurements from multiple passive sensors, such as the angle of arrival (AOA) [6,7]. Because of the mutual interference among multiple targets, data association becomes extremely important and essential.

However, the problem is especially complex and difficult. Because of the unknown corresponding relationship between measurements and targets, measurements from multiple passive sensors have to be first assigned to each possible target, which will lead to a combinatorial explosion as the number of senors and measurements increasing. Furthermore, in the presence of spurious measurements and missed detections of targets, a generalized association algorithm must allow for partial association and for unassigned measurements.

The traditional data association problem can generally be solved in the measurement domain with two steps. First, the data association problem is formulated as an *S*-dimensional (where *S* is the number of sensors) assignment problem [8,9,10], name as *S*DA problem. Then, the resulting *S*DA problem is solved by some optimal algorithms. However, the *S*DA problem is NP-hard for S ≥ 3 [11,12,13]. Therefore, the optimal solution algorithms, requiring an unacceptably long time, are of little practical value. Instead, suboptimal algorithms are more desirable.

One class of suboptimal algorithm to solve the *S*DA problem is the greedy heuristic algorithm. For example, the nearest neighbor (NN) heuristic algorithm [14] selects the assignment result with minimum cost in every loop. The tabu search algorithm [15] for the 3-D assignment problem introduces a so-called tabu list to store assignment results that are forbidden (i.e., tabu). The row-column algorithm [16,17] first arranges the cost vector of the *S*DA problem as a matrix and then finds the assignment result with the least value in a particular column.

Another alternative is the relaxation algorithm. Ref. [18] proposed a branch-and-band algorithm for the 3D assignment problem by using a Lagrangian relaxation. Refs. [19,20,21] developed a new iterative Lagrangian relaxation algorithm for the *S*DA problem. It introduced unconstrained Lagrangian multipliers to relax the *S*DA problem as a series of 2-D assignment subproblems, which can be solved in $O\left(n^{3}\right)$ (*n* is the number of measurements from each sensor) time using the modified auction algorithm [22,23].

These traditional *S*DA algorithms described above, solve the passive data association problem in the measurement domain. They follow the estimation and association steps to solve the problem. Estimation is to estimate all possible candidate target states by using measurements from all sensors. Association is the decision process of linking candidate targets of a common origin (true target). The performance of these algorithms is application condition related and the time complexity of these algorithms is at least $O\left(n^{S}\right)$. This is because that formulate the *S*DA problem needs to traverse all possible measurements to measurements combinations to estimate all possible candidate targets states, whose time complexity is $O\left(n^{S}\right)$. It typically takes only about 10% CPU time for solving the *S*DA problem when compared with the time for formulating the *S*DA problem [19]. Therefore, these *S*DA algorithms will be time-consuming when the number of sensors and targets are large.

To solve this problem, one obvious idea is to discard some false candidate targets when formulating the *S*DA problem, but it also takes extra time to decide which candidate target is false. Motivated by this fact, a new direct data assignment (DDA) algorithm is proposed in this paper. The major contributions and innovations of this paper can be concluded as follows:

- The passive data association problem is solved in the target state domain. The estimation process is replaced with assumed known candidate target states. The time complexity of the proposed DDA algorithm is linear with the number of sensors and targets. This means that the proposed DDA algorithm is more efficient compared with existing *S*DA algorithms when the number of sensors and targets are large.
- The number and states of the candidate targets are preset in the proposed DDA algorithm by the definition of region of interest. The costs and assignment results associated with the candidate targets are calculated by using the known states. Thus, the combinatorial explosion problem can be avoided.
- The number of candidate targets and measurements decreases as the number of iterations increases in the proposed DDA algorithm. This will make the time consumption for each iteration less and less.

The rest of this paper is organized as follows. We formulate the passive sensor data association problem in consideration in Section 2. The generalized *S*DA problem is discussed in Section 3. The proposed DDA algorithm is illustrated in Section 4. Simulation results and conclusions are given in Section 5 and Section 6 respectively.

## 2. Problem Formulation

**Figure 1:**
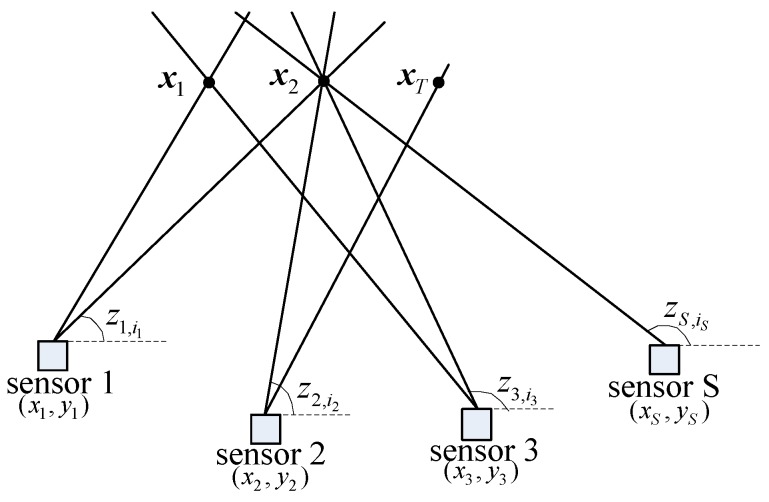
Passive sensor data association scenario.

The passive sensor data association scenario is shown in Figure 1. There are *T* (*T* unknown) targets and *S* bearings-only sensors in the region of interest. The positions of target *t* and sensor *s* are $x_{t}=\left(x_{t},y_{t}\right)$ and $x_{s}=\left(x_{s},y_{s}\right)$ respectively. We wish to associate the measurements from *S* sensors of $n_{s}$ measurements to decide which measurement came from which target. The AOA measurements from sensor *s* are $z_{si_{s}}$,

(1) $$z_{si_{s}}=\arctan\left(\frac{y_{t}-y_{s}}{x_{t}-x_{s}}\right)+v_{si_{s}}=h\left(x_{t},x_{s}\right)+v_{si_{s}},\;i_{s}=1,2,\ldots ,n_{s},$$

where $v_{si_{s}}$ is the additive measurement noise of sensor *s*. We assume that $v_{si_{s}}$ is Gaussian white noise with zero mean and covariance $\sigma_{s}^{2}$. Considering the missed detections, a target may not be detected by sensor *s*. We usually add dummy measurements $z_{s0}$ to each sensor. A dummy measurement from sensor *s* assigned to target $x_{t}$ means that this target was not detected by sensor *s*.

An *S*-tuple of measurements $\left\{z_{1i_{1}},z_{2i_{2}},\ldots ,z_{Si_{S}}\right\}$ can be built by taking a measurement from each sensor, and the likelihood that $\left\{z_{1i_{1}},z_{2i_{2}},\ldots ,z_{Si_{S}}\right\}$ originated from target *t*, with known target position $x_{t}$, is

(2) $$\begin{array}{c} f\left(z_{1i_{1}},z_{2i_{2}},\ldots ,z_{Si_{S}}|x_{t}\right)=\prod_{s=1}^{S}{\left[1-P_{D_{s}}\right]}^{1-u(i_{s})}\times {\left[P_{D_{s}}f\left(z_{si_{s}}|x_{t}\right)\right]}^{u(i_{s})}, \end{array}$$

where $P_{D_{s}}$ is the probability of detection of sensor *s*, $u(i_{s})$ is an indicator function, and $f(z_{si_{s}}|x_{t})$ is the probability density function of measurement $z_{si_{s}}$ originated from $x_{t}$,

(3) $$u\left(i_{s}\right)=\left\{\begin{array}{c} 0,\;\mathrm{if}\;i_{s}=0\\ 1,\;\mathrm{otherwise} \end{array}\right.$$

(4) $$f\left(z_{si_{s}}|x_{t}\right)=\frac{1}{\sqrt{2\pi }\sigma_{s}}\exp\left\{\frac{1}{2\sigma_{s}^{2}}{\left[z_{si_{s}}-h\left(x_{t},x_{s}\right)\right]}^{2}\right\}.$$

The likelihood that $\left\{z_{1i_{1}},z_{2i_{2}},\ldots ,z_{Si_{S}}\right\}$ are all spurious or unrelated to target $x_{t}$ is

(5) $$\begin{array}{c} f\left(z_{1i_{1}},z_{2i_{2}},\ldots ,z_{Si_{S}}|x_{t}=\phi \right)=\prod_{s=1}^{S}f\left(z_{si_{s}}|x_{t}=\phi \right)=\prod_{s=1}^{S}{\left[\frac{1}{V_{s}}\right]}^{u(i_{s})}, \end{array}$$

where $V_{s}$ is the field of view of sensor *s*.

The problem at hand now is to find the most likely set of *S*-tuples so that each measurement is assigned to at most one target or declared false, and each target receives at most one measurement from each sensor.

## 3. Generalized SDA Problem

The existing *S*DA algorithms solves the passive sensor data association problem by first reformulating it as an *S*DA problem, which is given by [19] as follows:

(6) $$\begin{array}{c} \min_{\rho_{i_{1}i_{2}\ldots i_{S}}}\sum_{i_{1}=0}^{n_{1}}\sum_{i_{2}=0}^{n_{2}}\ldots \sum_{i_{S}=0}^{n_{S}}c_{i_{1}i_{2}\ldots i_{S}}\rho_{i_{1}i_{2}\ldots i_{S}}, \end{array}$$

subject to

(7) $$\begin{array}{cc} \sum_{i_{2}=0}^{n_{2}}\sum_{i_{3}=0}^{n_{3}}\ldots \sum_{i_{S}=0}^{n_{S}}\rho_{i_{1}i_{2}\ldots i_{S}}=1; & \;i_{1}=1,2,\ldots ,n_{1}\\ \sum_{i_{1}=0}^{n_{1}}\sum_{i_{3}=0}^{n_{3}}\ldots \sum_{i_{S}=0}^{n_{S}}\rho_{i_{1}i_{2}\ldots i_{S}}=1; & \;i_{2}=1,2\ldots ,n_{2}\\ \;\;\vdots \;\;\vdots \;\;\vdots \\ \sum_{i_{1}=0}^{n_{1}}\sum_{i_{2}=0}^{n_{2}}\ldots \sum_{i_{S-1}=0}^{n_{S-1}}\rho_{i_{1}i_{2}\ldots i_{S}}=1; & \;i_{S}=1,2,\ldots ,n_{S} \end{array},$$

where $n_{s}$ is the number of measurements by sensor *s*. $\rho_{i_{1}i_{2}\ldots i_{S}}$ is a binary variable such that $\rho_{i_{1}i_{2}\ldots i_{S}}=1$ if the *S*-tuple $\left\{z_{1i_{1}},z_{2i_{2}},\ldots ,z_{Si_{S}}\right\}$ is included in the solution set. Otherwise, it is set to zero. $c_{i_{1}i_{2}\ldots i_{S}}$ is the cost of associating the *S*-tuple $\left\{z_{1i_{1}},z_{2i_{2}},\ldots ,z_{Si_{S}}\right\}$ to target $x_{t}$ which can be calculated as follows according to [24]:

(8) $$\begin{array}{cc} c_{i_{1}i_{2}\ldots i_{S}} & =-\ln\frac{f\left(z_{1i_{1}},z_{2i_{2}},\ldots ,z_{Si_{S}}|x_{t}\right)}{f\left(z_{1i_{1}},z_{2i_{2}},\ldots ,z_{Si_{S}}|x_{t}=\phi \right)}\\ \; & =\sum_{s=1}^{S}\left[u(i_{s})-1\right]\ln\left(1-P_{D_{s}}\right)-u\left(i_{s}\right)\ln\left(\frac{P_{D_{s}}V_{s}}{\sqrt{2\pi }\sigma_{s}}\right)+u\left(i_{s}\right)\frac{1}{2\sigma_{s}^{2}}{\left[z_{si_{s}}-h\left(x_{t},x_{s}\right)\right]}^{2}, \end{array}$$

where $x_{t}$ is usually replaced by its maximum likelihood estimate ${\hat{x}}_{t}$.

The resulting *S*DA problem described above is NP-hard for S≥3, which can be solved by using the greedy heuristic algorithm or relaxation algorithm. It can be seen from (6) that the operation involved in constructing the *S*DA problem is $O(n^{S}c_{0})$, where *n* is the average number of measurements from each sensor and $c_{0}$ is the average time used to compute one cost of the *S*-tuple in (8). Therefore, the time complexity of the generalized *S*DA algorithms are at least $O(n^{S}c_{0})$, which is exponential with the number of targets and sensors.

Besides, as the actual position $x_{t}$ in (8) is unknown, it is usually replaced by the maximum likelihood estimate. This is a nonlinear optimization problem and can be solved by the iterative least squares (ILS) algorithm [25]. However, the ILS algorithm may converge to a local minimum solution when the measurement noise is at a high level, which can deteriorate the performance of the association results.

## 4. Linear-Time DDA Algorithm

The goal of passive sensor data association is to find the correct set of *S*-tuples $\left\{z_{1i_{1}},z_{2i_{2}},\ldots ,z_{Si_{S}}\right\}$ such that all measurements from the same target are in the same *S*-tuple. The correct *S*-tuples are then used to determine the positions of the targets. Since the number and the positions of the targets are unknown, the generalized *S*DA algorithms follow the estimation and association steps to solve the problem. Estimation is to estimate all possible candidate targets and costs, which has a time complexity of $O(n^{S})$. Association is the decision process of linking candidate targets of a common origin (true target).

It can be seen that the main reason for the high time complexity of the *S*DA algorithms is that the passive sensor data association problem is solved in the measurement domain. In contrast, the proposed DDA algorithm described below solves the association problem in the target state domain by first presetting candidate targets with known positions in the region of interest and then calculating the costs and *S*-tuples associated with the candidate targets. The assignment results are obtained by finding the candidate target with the minimum cost. It will be found that the time complexity of the proposed DDA algorithm is linear with the number of sensors and targets.

### 4.1. Mechanism of DDA Algorithm

**Figure 2:**
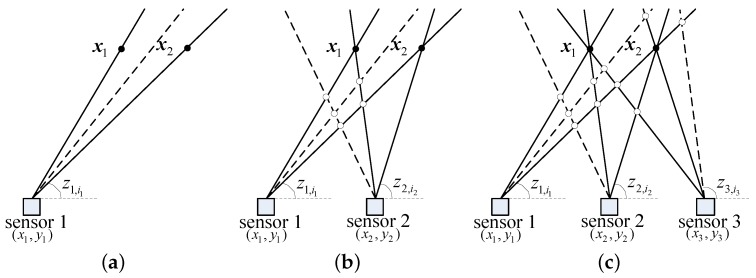
Mechanism of the direct data assignment (DDA) algorithm. (**a**) One sensor to detect targets. (**b**) Two sensors to detect targets. (**c**) Three sensors to detect targets. **—** true measurement, **- - -** spurious measurement, • true target, ∘ false target.

Without loss of generality, it is assumed that there are two targets whose positions are $x_{1}$ and $x_{2}$ in the region of interest. Each sensor has a detection probability of $P_{D}=1$ and one spurious measurement per scan. If we have only one sensor to detect the targets, as shown in Figure 2a, only one measurement is assigned to the true target. We can just get the AOA measurements of the two targets.

If there are two sensors, as shown in Figure 2b, two true measurements from the two sensors are assigned to one of the true targets and then the position can be obtained using the triangulation algorithm [26]. Unfortunately, spurious and true measurements will also be assigned to some false targets. Thus, the true targets and false targets can not be distinguished if there is no prior information.

When the number of sensors increases to three, as shown in Figure 2c, three true measurements are assigned to the true target and only two spurious and true measurements are assigned to the false target. Therefore, the true target can be distinguished according to the number of assigned measurements.

As the number of sensors increases, the difference between true targets and false targets becomes more and more obvious.

However, using the number of assigned measurements to distinguish the true targets from the false targets may be inappropriate because of the the measurement noise. To overcome this problem, we can first define a cost function $c_{tsi_{s}}$ (similar to (8)) to evaluate the cost of the the $i_{s}$th measurement of sensor *s* assigned to target $x_{t}$, and then choose the $i_{s}^{*}$-th measurement with the minimum cost $c_{tsi_{s}^{*}}$. For *S* sensors, the *S*-tuple assigned to $x_{t}$ is $\left\{z_{1i_{1}^{*}},z_{2i_{2}^{*}},\ldots ,z_{Si_{S}^{*}}\right\}$, and the total cost of $x_{t}$ is

(9) $$\begin{array}{c} \varphi_{t}=\sum_{s=1}^{S}c_{tsi_{s}^{*}}, \end{array}$$

with

(10) $$\begin{array}{c} i_{s}^{*}=\arg\min_{i_{s}}c_{tsi_{s}},\;i_{s}=0,1,2,\ldots n_{s}. \end{array}$$

(11) $$\begin{array}{cc} c_{tsi_{s}} & =-\ln\frac{{\left[1-P_{D_{s}}\right]}^{1-u_{1}\left(i_{s}\right)}\times {\left[P_{D_{s}}f\left(z_{si_{s}}|x_{t}\right)\right]}^{u_{1}\left(i_{s}\right)}}{f\left(z_{si_{s}}|x_{t}=\phi \right)}\\ \; & =\left[u_{1}\left(i_{s}\right)-1\right]\ln\left(1-P_{D_{s}}\right)-u_{1}\left(i_{s}\right)\ln\left(\frac{P_{D_{s}}V_{s}}{\sqrt{2\pi }\sigma_{s}}\right)+u_{1}\left(i_{s}\right)\frac{1}{2\sigma_{s}^{2}}{\left[z_{si_{s}}-h\left(x_{t},x_{s}\right)\right]}^{2}, \end{array}$$

(12) $$u_{1}\left(i_{s}\right)=\left\{\begin{array}{c} 0,\;\mathrm{if}\;i_{s}=0\;\mathrm{or}\;\left|z_{si_{s}}-h\left(x_{t},x_{s}\right)\right|>T_{0}\\ 1,\;\mathrm{otherwise} \end{array}\right.,$$

where $T_{0}$ is the measurement threshold, which can usually be set to $3\sigma_{s}$. It can be seen that the measurement threshold $T_{0}$ is determined by the noise level of each sensor. When the noise level is high, the threshold is relatively large. Thus, measurements with large noise can also be assigned to the target. Besides, using the threshold can also abandon false measurements or measurements from other targets that are too far from the target. In this way, the mutual influence of measurements between the targets can be avoid. This will make the proposed algorithm still effective in low detection probability and high clutter density conditions.

According to (9), (11) and (12), the true targets can be easily distinguished from the false targets, since the true targets has a cost less than the false targets. The cost of false targets can be calculated through the false targets positions in (11) and (12).

**Figure 3:**
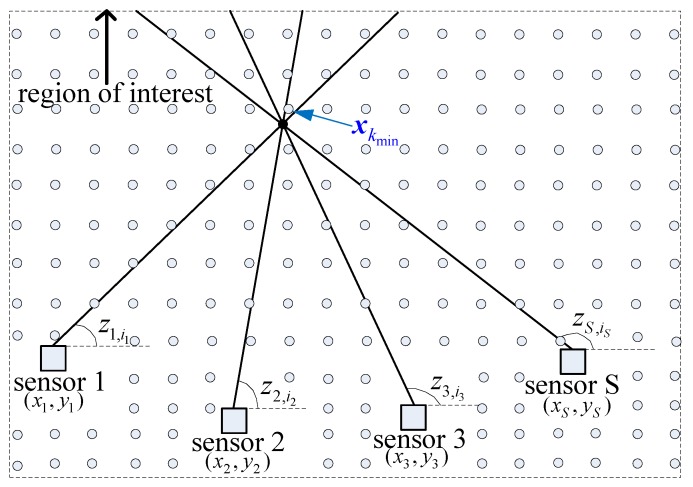
Mechanism of the DDA algorithm. **—** true measurement, • true target, ∘ candidate target.

The position of target $x_{t}$ is usually unknown in reality. However, the region of interest (i.e., all the detected targets are in this region) is generally known, as shown in Figure 3. *K* candidate targets with known positions $x_{k},k=1,2,\ldots ,K$ can be preset in the region of interest, and then the cost in (9) is calculated using the candidate position $x_{k}$. Thus, each candidate target corresponds to one association hypothesis. The candidate position $x_{k_{\min}}$ with the minimum cost and desired associated *S*-tuple $\left\{z_{1i_{1}^{*}},z_{2i_{2}^{*}},\ldots ,z_{Si_{S}^{*}}\right\}$ is obtained by traversing all candidate positions. The passive sensor data association problem is converted to a linear minimization problem in the target state domain. If there are multiple targets, we need to execute the above operations iteratively until all the measurements are assigned to a candidate target or some prespecified threshold.

In addition, under the constraint that each measurement is assigned to at most one target or declared false, the assigned measurements after every iteration must be deleted from the measurement lists. Details of the DDA algorithm are shown in Algorithm 1.

From Algorithm 1, it can be found that the operations involved in each iteration are as follows: (1) $nSKc_{1}$ operations for computing all possible costs $c_{ksi_{s}}$ in step 2, where $c_{1}$ is the average time used to compute one candidate cost using (11) and *n* is the average number of measurements from each sensor. (2) nS operations for computing $\varphi_{k}$ in step 3, (3) K+S operations for finding the candidate with minimum cost and delete related measurements in step 4.

Thus, the time complexity of the proposed DDA algorithm is $O\left(nSKm_{1}c_{1}\right)$ ($m_{1}$ is the number of iterations), which is linear with the number of sensors and targets.

**Algorithm 1.** (DDA algorithm).
**Step 1—initialization:**
Construct the candidate target positions in the region of interest,$x_{k}=\left(x_{k},y_{k}\right),k=1,2,\ldots K$;   $\hat{N}=0$;    Total cost Λ=0.
**Step 2—compute all possible costs $c_{ksi_{s}}$:**
   **do**
$\forall k,s,i_{s}$   $c_{ksi_{s}}$ is computed using (11) through candidate target $x_{k}$.   **end do**
**Step 3—compute candidate cost and associated *S*-tuple:**
   **do**
∀k   $\varphi_{k}=\sum_{s=1}^{S}c_{ksi_{s}^{*}};\gamma_{k}=\left\{z_{1i_{1}^{*}},z_{2i_{2}^{*}},\ldots ,z_{Si_{S}^{*}}\right\}$.   where $i_{s}^{*}=\arg\min_{i_{s}}c_{ksi_{s}},i_{s}=0,1,2,\ldots n_{s}$.   **end do**
**Step 4—find candidate association with minimum cost and iteration:**

$k_{\min}=\arg\min_{k}\varphi_{k}$
if $\varphi_{k_{\min}}>0$; go to **Step 5**$\hat{N}=\hat{N}+1$;    $d_{\hat{N}}=\gamma_{k_{\min}}=\left\{z_{1i_{1}^{*}},z_{2i_{2}^{*}},\ldots ,z_{Si_{S}^{*}}\right\}$;    $\Lambda =\Lambda +\varphi_{k_{\min}}$.**Delete** related $\left\{z_{1i_{1}^{*}},z_{2i_{2}^{*}},\ldots ,z_{Si_{S}^{*}}\right\}$ from measurement lists.Go to **Step 2**.
**Step 5—final result:**
   Number of Targets $=\hat{N}$;   *S*-tuples $=d=\{d_{k};\;k=0,1,\ldots \hat{N}\}$;   Total cost Λ=Λ.

### 4.2. Termination Condition

It can be seen from Algorithm 1 that the *S*-tuple assigned to candidate target $x_{k}$ is $\gamma_{k}\;=\;\left\{z_{1i_{1}^{*}},z_{2i_{2}^{*}},\ldots ,z_{Si_{S}^{*}}\right\}$. The likelihood that $\left\{z_{1i_{1}^{*}},z_{2i_{2}^{*}},\ldots ,z_{Si_{S}^{*}}\right\}$ originated from $x_{k}$ is

(13) $$\begin{array}{c} f\left(z_{1i_{1}^{*}},z_{2i_{2}^{*}},\ldots ,z_{Si_{S}^{*}}|x_{k}\right)=\prod_{s=1}^{S}{\left[1-P_{D_{s}}\right]}^{1-u_{1}\left(i_{s}^{*}\right)}\times {\left[P_{D_{s}}f\left(z_{si_{s}}^{*}|x_{k}\right)\right]}^{u_{1}\left(i_{s}^{*}\right)}. \end{array}$$

The likelihood that $\left\{z_{1i_{1}^{*}},z_{2i_{2}^{*}},\ldots ,z_{Si_{S}^{*}}\right\}$ are all spurious is

(14) $$\begin{array}{c} f\left(z_{1i_{1}^{*}},z_{2i_{2}^{*}},\ldots ,z_{Si_{S}^{*}}|x_{k}=\phi \right)=\prod_{s=1}^{S}{\left[\frac{1}{V_{s}}\right]}^{u(i_{s}^{*})}. \end{array}$$

The negative log-likelihood ratio [24] can be defined as

(15) $$\begin{array}{cc} -\ln\frac{f\left(z_{1i_{1}^{*}},z_{2i_{2}^{*}},\ldots ,z_{Si_{S}^{*}}|x_{k}\right)}{f\left(z_{1i_{1}^{*}},z_{2i_{2}^{*}},\ldots ,z_{Si_{S}^{*}}|x_{k}=\phi \right)} & =\sum_{s=1}^{S}-\ln\frac{{\left[1-P_{D_{s}}\right]}^{1-u_{1}\left(i_{s}^{*}\right)}\times {\left[P_{D_{s}}f\left(z_{si_{s}^{*}}|x_{k}\right)\right]}^{u_{1}\left(i_{s}\right)}}{f\left(z_{si_{s}^{*}}|x_{k}=\phi \right)}\\ \; & =\sum_{s=1}^{S}c_{ksi_{s}^{*}}=\varphi_{k}. \end{array}$$

If $\varphi_{k}>0$, the probability of $\left\{z_{1i_{1}^{*}},z_{2i_{2}^{*}},\ldots ,z_{Si_{S}^{*}}\right\}$ originated from $x_{k}$ is smaller than the probability of $\left\{z_{1i_{1}^{*}},z_{2i_{2}^{*}},\ldots ,z_{Si_{S}^{*}}\right\}$ are all spurious. In this case, the *S*-tuple $\left\{z_{1i_{1}^{*}},z_{2i_{2}^{*}},\ldots ,z_{Si_{S}^{*}}\right\}$ may generate a false target.

Besides, $\varphi_{k}$ will be updated at **Step 2** and **Step 3** of Algorithm 1 in every iteration. According to (9), (10) and (11), it can be found that

(16) $$\begin{array}{c} \varphi_{k}^{\left(l_{1}\right)}\leq \varphi_{k}^{\left(l_{2}\right)},\;\mathrm{if}\;l_{1}\leq l_{2}, \end{array}$$

where $l_{1}$ and $l_{2}$ are the numbers of iterations in Algorithm 1. This is because that measurements assigned to candidate $x_{k_{\min}}$ will be deleted in every iteration, and less measurements may makes a bigger $c_{ksi_{s}^{*}}$ with a bigger $\varphi_{k}$.

Therefore, the iteration of Algorithm 1 can be terminated if $\varphi_{k_{\min}}>0$, and the total cost Λ in Algorithm 1 is minimum. Besides, candidate targets with $\varphi_{k}>0$ can be deleted in the process of iteration. Thus, the number of candidate targets and measurements decreases as the number of iterations increases, and the running time for each iteration will reduce.

### 4.3. Performance Analysis

In this subsection, we analyze the performance of the proposed DDA algorithm and the SDA algorithm. It can be seen from (15) and (8) that the cost of candidate target $x_{k}$ in the proposed DDA algorithm is similar to that in the *S*DA algorithm. However, the difference is obvious.

In the generalized *S*DA algorithm, the passive sensor measurements assignment problem is solved in the measurement domain. The position of candidate target $x_{t}$ is unknown before the association process. It can only be estimated after obtaining the associated *S*-tuple $\left\{z_{1i_{1}},z_{2i_{2}},\ldots ,z_{Si_{S}}\right\}$, and then the cost is calculated. The time complexity of this process is $O(n^{S})$, which is exponential with *n* and *S*. Besides, the candidate position estimate ${\hat{x}}_{t}$ may be inaccurate when the measurement noise is at a high level, which will deteriorate the performance of the algorithm.

However, in the proposed DDA algorithm, the passive sensor measurements assignment problem is solved in the target state domain. The position of candidate target $x_{k}$ is assumed known before the association process. The assumed known $x_{k}$ is used to find the associated *S*-tuple $\left\{z_{1i_{1}^{*}},z_{2i_{2}^{*}},\ldots ,z_{Si_{S}^{*}}\right\}$, and calculate the cost accordingly. The time complexity of this process is O(nSK), which is linear with *n* and *S*.

Therefore, the proposed DDA algorithm is more efficient than the *S*DA algorithm when the number of sensors and targets are large. Besides, because the candidate target position $x_{k}$ is assumed known in the proposed DDA algorithm, the measurement threshold can be used in (12) to abandon measurements that are far from the candidate target.

**Figure 4:**
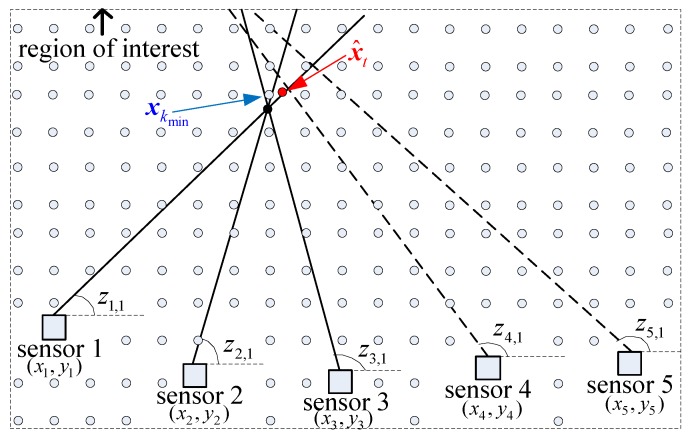
Threshold to abandon measurements far from the candidate target. **—** true measurement, **- - -** spurious measurement, • true target, ∘ candidate target.

For example, there are five sensors and one target in the region of interest, as shown in Figure 4. Senors 1–3 detect the target signal and generate three measurements $z_{1,1}$, $z_{2,1}$ and $z_{3,1}$. Sensors 4 and 5 have missed detections and two spurious measurements $z_{4,1}$ and $z_{5,1}$, with $\left|z_{si_{s}}-h\left(x_{k_{\min}},x_{s}\right)\right|>T_{0}$ for s=4,5. The *S*-tuple result of the proposed DDA algorithm is $\left\{z_{1,1},z_{2,2},z_{3,1},z_{4,0},z_{5,0}\right\}$. While for *S*DA algorithm, the the cost of *S*-tuple $\left\{z_{1,1},z_{2,2},z_{3,1},z_{4,0},z_{5,0}\right\}$ may be bigger than the cost of *S*-tuple $\left\{z_{1,1},z_{2,2},z_{3,1},z_{4,1},z_{5,1}\right\}$ in some cases. So the output *S*-tuple result is $\left\{z_{1,1},z_{2,2},z_{3,1},z_{4,1},z_{5,1}\right\}$.

### 4.4. Initialization of Candidate Targets

It can be observed from Figure 4 that the main factors affecting the performance of the proposed DDA algorithm is the number of candidate targets. If we have enough candidate targets in the region of interest, *T* (*T* is the number of true targets) candidate targets will be very close to the true targets. The true *S*-tuples of measurements associated with the *T* targets can be easily obtained in this case. However, the time complexity of the proposed DDA algorithm is linear with the number of candidate targets. More candidate targets lead to more time consumption.

There are many algorithms that can be used to initialize the positions of candidate targets, such as the multiple grid algorithms [27,28], particle swarm optimization (PSO) algorithms [29], etc. Here, we use the simple grid-based algorithm [30]. The grid spacing $d_{0}$, which is the distance between each two candidate targets, is related to the Cramér–Rao lower bound (CRLB) [31] of localization error.

The CRLB is usually used to evaluate the variance lower bound of any unbiased estimator [32]. This means that two targets are theoretically indistinguishable when the distance between them is less than two times of the CRLB. To be more secure, the grid spacing $d_{0}$ can be approximately set to one time of the CRLB,

(17) $$\begin{array}{c} d_{0}=\sqrt{\mathrm{tr}\left(\mathrm{CRLB}(x_{k})\right)} \end{array}$$

where tr(∗) denotes the trace of a matrix. The derivation of $\mathrm{CRLB}(x_{k})$ is given in Appendix A.

**Figure 5:**
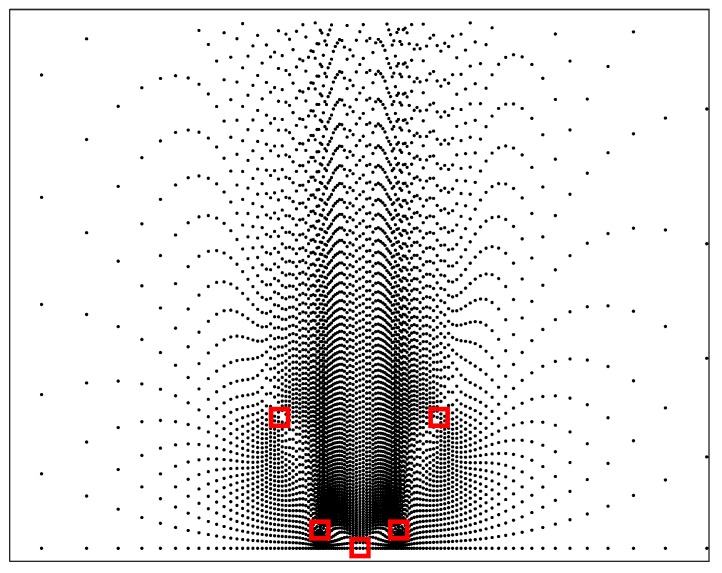
Geometry of candidate targets. □ sensor, · candidate target.

In this way, the grid spacing $d_{0}$ strictly depends on the position of the candidate target which is known in advance. In practice, we can randomly select an initial candidate target in the region of interest, and the position of the next candidate target is determined by the CRLB of the current candidate target, as shown in Figure 5. It can be seen that the closer to the sensors, the higher the density of candidate targets. This is because the CRLB is smaller when the candidate target is closer to the sensors.

**Figure 6:**
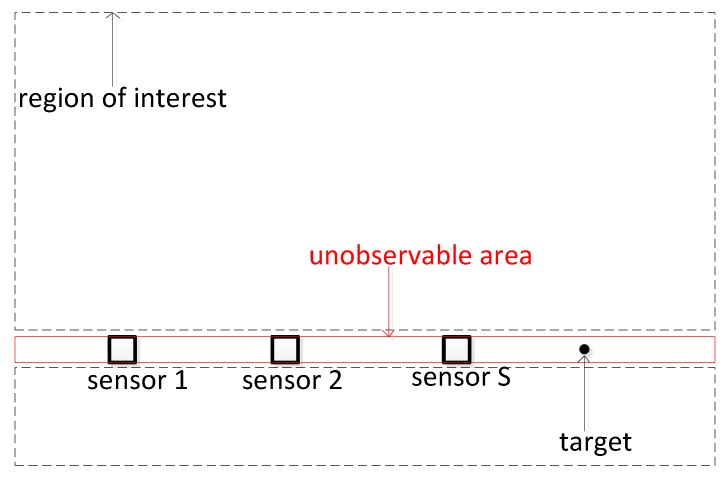
Geometry of unobservable area and region of interest.

Besides, the system observability [33,34] may affect the performance of the proposed algorithm. For example, if the sensors and targets are distributed on a line, as shown in Figure 6, the position of the target is theoretically unavailable.

In order to avoid producing incorrect assignment results from the absence of system observability, a feasible solution is to first remove the unobservable area from the region of interest in our proposed algorithm. Thus, the wrong assignment results can be avoided in the absence of observability, as shown Figure 6.

## 5. Computer Simulation

**Figure 7:**
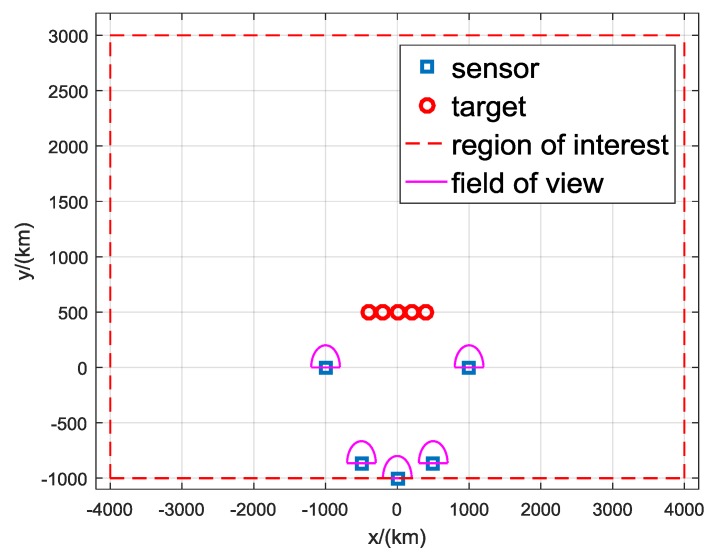
Geometry used in simulation.

The simulation scenario is shown in Figure 7. There are *S* sensors at known fixed locations in a semicircle of radius 1000 km, and the position of sensor *s* is $-1000\left(\cos\left(\frac{s-1}{S-1}\pi \right),\sin\left(\frac{s-1}{S-1}\pi \right)\right)$ km, s=1,2,…,S. The targets are symmetrically distributed on the *x*-axis with y=500 km, different *x*. The region of interest is the area with x∈[−4000,4000] km and y∈[−1000,3000] km. The candidate targets of the proposed DDA algorithm are uniformly distributed in the region of interest. The sensors are assumed to be forward looking with a field of view of $0∼180^{\circ }$. The detection probability of each sensor is $P_{D}=0.9$.

The performance of the proposed DDA algorithm is compared with that of two kinds of *S*DA algorithms, the row–column algorithm in [16] and the Lagrangian relaxation algorithm in [19]. The positions and costs of the candidate targets in the *S*DA algorithms are estimated by using the ILS algorithm. Estimated candidate targets with a cost greater than zero are first deleted in the process of forming the *S*DA problem [19].

Six typical cases are investigated in this section. The first is used to test the performance of proposed algorithm for different grid spacing. The second is carried out for different numbers of sensors. The third is done for different number of targets. The fourth is simulated for different measurement noise levels. The fifth is done in different detection probabilities. The last one is simulated in a challenge scenario. Three kinds of scenarios, normal, high clutter and poor separated, are performed. Details of the three scenarios are shown in Table 1. The number of spurious measurements for each sensor is 0.8π≈2.5 in the normal scenario, and 1.5π≈4.7 in the high clutter scenario. The distance between each two targets is about 20% of the distance between sensors and targets in the normal scenario, which is about 4% in the poor separated scenario.

The association accuracy *p* and root mean square error (RMSE) [32] are computed over N=200 ensemble runs, which are defined as follows.

(18) $$p=\frac{\sum_{i=1}^{N}n_{r}^{i}}{\sum_{i=1}^{N}n_{A}^{i}},$$

(19) $$RMSE=\sqrt{\frac{1}{N}\sum_{i=1}^{N}{∥{\hat{x}}_{t}^{i}-x_{t}∥}^{2}},$$

where $n_{r}^{i}$ and $n_{A}^{i}$ are the number of correct associated measurements and all measurements, and ${\hat{x}}_{t}^{i}$ is the target position estimate at ensemble *i*.

The positions of the targets are estimated by using the assignment results. If the association accuracy is low, the estimated positions is far away from positions of the true targets, and the RMSE is relatively large. Besides, a lower association accuracy means more false assignment results, which lead to a larger number of false targets.

### 5.1. Simulation Results of Case 1

The performance affected by the grid spacing was studied in this case. There were five sensors and five targets in the simulation. The measurement noise standard deviation of all sensors were equal to $\sigma =0.5^{\circ }$ and the grid spacing is from $0.25d_{0}$ to $4d_{0}$.a

Simulation results of normal scenario is shown in Table 2. It can be seen that the association accuracy was the same when grid spacing was less than $2d_{0}$, but the time consumption decreased with the increase of grid spacing.

Table 3 and Table 4 illustrate the results of high clutter and poor separated scenario respectively. The conclusions were consistent with that in the normal scenario. When the grid spacing is less than $2d_{0}$, reducing the grid spacing has no effect on the association accuracy of the proposed DDA algorithm, but it will increase the time consumption.

The number of spurious measurements in a high clutter scenario was more than that in the normal scenario, which led to more false intersections and computations. Thus, the association accuracy was lower, while the average run time was greater, as shown in Table 3. Besides, poor separated targets result in a lower measurement discrimination, which leads to a lower association accuracy, as shown in Table 4.

### 5.2. Simulation Results of Case 2

The performance affected by the number of sensors was studied in this case. Three kinds of scenarios were simulated with 3, 5 and 7 sensors. The number of targets was five and the measurement noise standard deviation was equal to $\sigma =0.5^{\circ }$. The grid spacing was $d_{0}$.

Simulation results of the normal scenario is shown in Table 5. Evidently, increasing the number of sensors can increase the association accuracy, but it also leads to an increase in time consumption. The association accuracy of the proposed algorithm was almost the same with the relaxation algorithm but a little superior to the row–column algorithm. We think the reason for a little higher association accuracy may be that the threshold has been used in the proposed algorithm. Thus, the spurious measurements that are too far from the candidate targets can be abandoned, which may improve the association accuracy, as shown in Figure 4. The average run time of the proposed algorithm increased from 1.1 s with three sensors to 4.2 s with seven sensors. However, the time consumption of the relaxation algorithm and the row–column algorithm with seven sensors were 407 and 386 s respectively. This is because that the time complexity of the proposed algorithm is linear with the number of sensors while that of the relaxation algorithm and row–column algorithm are exponential.

In Table 6, simulation results of high clutter scenario are presented. The time consumption superiority of the proposed algorithm can still be maintained in this scenario. The average run time of the proposed algorithm with seven sensors was 4.6 s. However, the relaxation algorithm had an average run time of 2736 s with seven sensors.

Table 7 shows simulation the results of poor separated scenario. It can be seen that the association accuracy of the proposed algorithm is similar to that of the relaxation algorithm and row–column algorithm, but the average run time of the proposed algorithm was significantly less. Interestingly, the association accuracy cannot be significantly improved by increasing the number of sensors. This is because that the distance between every two targets is too small, the measurements come from different targets that are indistinguishable.

### 5.3. Simulation Results of Case 3

The performance affected by the number of targets was studied in this case. Three kinds of scenarios were simulated with 5, 7 and 9 targets. The number of sensors was five and the measurement noise standard deviation was equal to $\sigma =0.5^{\circ }$. The grid spacing is $d_{0}$.

Simulation results of normal scenario are shown in Table 8. It can be seen that the association accuracy decreases as the number of targets increases. This is because more targets generate more measurements, resulting in more measurement interactions and ghosts targets. The association accuracy of the proposed algorithm was still almost the same with the relaxation algorithm but superior to the row–column algorithm. The average run time of the proposed algorithm was still less than that of the relaxation algorithm and arow column algorithm.

Table 9 and Table 10 show the results of high clutter and poor separated scenarios respectively. The conclusions were consistent with the normal scenario. Since more spurious measurements were generated with higher clutter density, the average run time of high clutter scenario was more than that of normal scenario, and the association accuracy is relatively lower, as shown in Table 9. Since the targets were undistinguishable in poor separated scenario, the three algorithms had almost the same association accuracy, as shown in Table 10.

### 5.4. Simulation Results of Case 4

To further illustrate the advantage of the proposed algorithm, a comparison between different measurement noise levels was made. This simulation contained five sensors and five targets. The measurement noise standard deviations of all sensors were $\sigma \in \{0.5^{\circ },1^{\circ },1.5^{\circ }\}$, and the grid spacing was $d_{0}$.

Simulation results of the normal scenario are shown in Table 11. An obvious trend is that the large standard deviation of noise resulted in unreliable data association because a large σ may cause the measured value to deviate farther from the real one, creating more ghosts. The association accuracy of the proposed algorithm was still almost the same with the relaxation algorithm and higher than the row column algorithm. Because the CRLB increased as the σ increased, the grid spacing $d_{0}$ also increased. So the number of candidate targets in the proposed algorithm decreased, resulting in a lower run time.

In Table 12 and Table 13, we present the simulation results of high clutter scenario and poor separated scenario. High clutter density led to a lower association accuracy and more number of false targets. Interestingly, the RMSE in poor separated scenario with $\sigma =1^{\circ }$ and $\sigma =1.5^{\circ }$ are smaller than those in normal scenario. The reason is that measurements came from adjacent targets are undistinguishable in poor separated scenario. When the measurement noise standard deviation was large, the localization error caused by the measurement noise became larger than the distance from each two targets.

### 5.5. Simulation Results of Case 5

To further show the boundary of the proposed algorithm, a comparison between different detection probabilities was made. This simulation contained five sensors and five targets. The detection probability of all sensors are $P_{D}\in \left\{0.9,0.7,0.5\right\}$. The measurement noise standard deviation was equal to $\sigma =0.5^{\circ }$, and the grid spacing was $d_{0}$.a

Simulation results are shown in Table 14. One obvious trend is that the lower detection probability results in lower association accuracy because a lower $P_{D}$ makes fewer true measurements, creating fewer true targets. The association accuracy of the proposed algorithm was still almost the same with the relaxation algorithm and higher than the row–column algorithm. Because the number of measurements decreased as the $P_{D}$ decreased, the average run time was also decreased.

### 5.6. Simulation Results of Case 6

**Figure 8:**
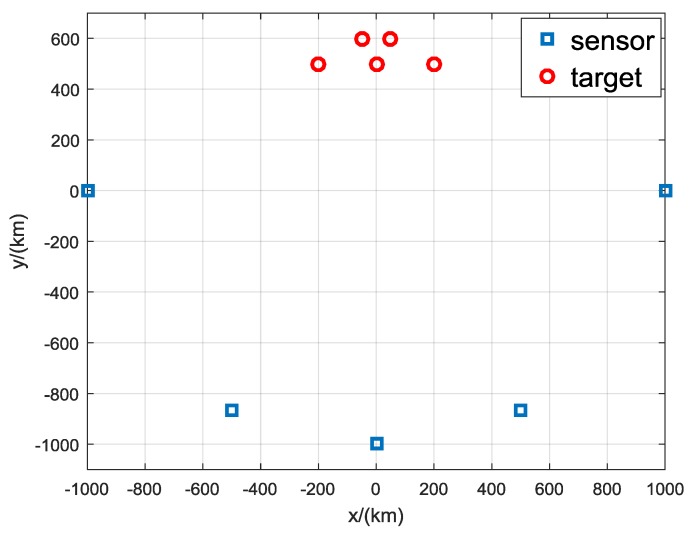
Geometry of a more challenging scenario.

To further verify the performance of the proposed algorithm under challenge scenarios, a comparison with targets was not separated in AOA measurements is made. This simulation contains five sensors with the same positions as noted above. There are five targets with the positions of $x_{1}\left(-200,500\right)$ km, $x_{2}\left(0,500\right)$ km, $x_{3}\left(200,500\right)$ km, $x_{4}\left(-50,600\right)$ km, $x_{5}\left(50,600\right)$ km, as shown in Figure 8. The true AOA measurements of the five targets measured by the five sensors are shown in Table 15. It can be seen that some the five targets are very poor separated in AOA measurements. The clutter density is 0.8/rad and the detection probability $P_{D}$ is 0.9. Simulations are done with different measurements noise levels $\sigma \in \{0.5^{\circ },1^{\circ },1.5^{\circ }\}$.

Simulation results are shown in Table 16. It can be seen that the association accuracy decreases as the noise levels increase, which is the same as Case 4. The association accuracy of the proposed algorithm is still almost the same with the relaxation algorithm and higher than the row–column algorithm. One may realize that the association accuracy in Table 16 is relatively lower than that in Table 11. This is because some of the targets were not separated in AOA measurements, which will generate some unresolved targets.

## 6. Conclusions

To solve the problem of passive sensor data association, a linear-time DDA algorithm is proposed in this paper. Different from existing algorithms that solve the problem in the measurement domain, the proposed DDA algorithm solves the problem directly in the target state domain. The number and state of candidate targets are preset by the definition of region of interest, which can avoid the problem of combinational explosion. The time complexity of the proposed DDA algorithm is linear with the number of sensors and targets while that of the existing algorithms is exponential. Since the positions of the preset candidate targets are known, the threshold can be used to abandon spurious measurements that are far from the candidate targets. Simulations are performed with three kinds of scenarios; normal, high clutter, and poor separation, which show that the proposed DDA algorithm has a significantly lower run time and can achieve almost the same association accuracy as existing algorithms. 

## Figures and Tables

**Table 1:** Parameters of three scenarios.

Scenario	Clutter Density	Distance between Targets
Normal	0.8/rad	200 km
High clutter	1.5/rad	200 km
Poor separated	0.8/rad	40 km

**Table 2:** Simulation results of normal scenario with different grid spacing.

Grid Spacing/$d_{0}$	0.25	0.5	1	2	4
Association accuracy	93%	93%	93%	93%	91%
Average run time (s)	41.3	9.7	2.4	0.6	0.1
Average number of false targets	1.2	1.2	1.2	1.2	1.4
RMSE (km)	11.3	11.3	11.3	11.4	11.9

**Table 3:** Simulation results of high clutter scenario with different grid spacing.

Grid Spacing/$d_{0}$	0.25	0.5	1	2	4
Association accuracy	86%	86%	86%	85%	80%
Average run time (s)	51.4	11.7	2.8	0.7	0.2
Average number of false targets	3	3	3	3.1	3.3
RMSE (km)	12.2	12.3	12.2	12.2	12.3

**Table 4:** Simulation results of poor separated scenario with different grid spacing.

Grid Spacing/$d_{0}$	0.25	0.5	1	2	4
Association accuracy	57%	57%	57%	57%	54%
Average run time (s)	29.7	7.2	2.0	0.5	0.1
Average number of false targets	1.5	1.5	1.5	1.5	1.7
RMSE (km)	20.3	20.3	20.3	20.3	20.5

**Table 5:** Simulation results of normal scenario with different number of sensors.

	Proposed Algorithm			Relaxation Algorithm			Row Column Algorithm		
Number of sensors	3	5	7	3	5	7	3	5	7
Association accuracy	42%	93%	98%	41%	94%	97%	40%	87%	92%
Average run time (s)	1.1	2.4	4.2	0.1	9.2	407	0.1	7.3	386
Average number of false targets	4.6	1.2	0.1	4.6	1	0.1	4.8	1.3	0.4
RMSE (km)	16.8	11.3	9.6	16.8	11	9.6	16.8	11.5	10.8

**Table 6:** Simulation results of high clutter scenario with different number of sensors.

	Proposed Algorithm			Relaxation Algorithm			Row Column Algorithm		
Number of sensors	3	5	7	3	5	7	3	5	7
Association accuracy	38%	86%	95%	37%	85%	94%	35%	82%	90%
Average run time (s)	1.3	2.8	4.6	0.3	40.2	2736	0.3	28.5	2357
Average number of false targets	6.4	3.0	1.1	6.6	3.1	1.2	7.3	3.3	1.7
RMSE (km)	17.8	12.2	9.9	18.0	12.2	10.0	18.3	12.8	11.0

**Table 7:** Simulation results of poor separated scenario with different number of sensors.

	Proposed Algorithm			Relaxation Algorithm			Row Column Algorithm		
Number of sensors	3	5	7	3	5	7	3	5	7
Association accuracy	32%	57%	57%	32%	57%	57%	31%	56%	56%
Average run time (s)	1.0	2.3	3.6	0.1	9.8	396	0.1	7.5	375
Average number of false targets	5.1	1.5	0.9	5.2	1.5	0.9	5.2	1.7	1.1
RMSE (km)	21.7	20.3	18.0	21.6	20.3	18.0	22.0	20.5	18.4

**Table 8:** Simulation results of normal scenario with different number of targets.

	Proposed Algorithm			Relaxation Algorithm			Row Column Algorithm		
Number of targets	5	7	9	5	7	9	5	7	9
Association accuracy	93%	87%	85%	94%	87%	85%	87%	83%	80%
Average run time (s)	2.4	3.0	3.5	9.2	39.1	70.7	7.3	27.3	60.5
Average number of false targets	1.2	1.5	1.7	1.0	1.5	1.7	1.3	1.7	1.9
RMSE (km)	11.3	12.1	12.3	11.0	12.0	12.2	11.5	12.3	12.8

**Table 9:** Simulation results of high clutter scenario with different number of targets.

	Proposed Algorithm			Relaxation Algorithm			Row Column Algorithm		
Number of targets	5	7	9	5	7	9	5	7	9
Association accuracy	86%	81%	76%	85%	81%	77%	82%	76%	71%
Average run time (s)	2.8	3.7	4.6	40.2	92.5	135.3	28.5	64.0	124.0
Average number of false targets	3.0	3.3	3.9	3.1	3.3	3.8	3.3	3.9	4.7
RMSE (km)	12.2	12.7	13.3	12.2	12.5	13.2	12.8	13.0	13.6

**Table 10:** Simulation results of poor separated scenario with different number of targets.

	Proposed Algorithm			Relaxation Algorithm			Row Column Algorithm		
Number of targets	5	7	9	5	7	9	5	7	9
Association accuracy	57%	47%	41%	57%	47%	41%	56%	47%	39%
Average run time (s)	2.3	2.9	3.3	9.8	41.3	80.6	7.5	19.1	70.9
Average number of false targets	1.5	2.4	3.6	1.5	2.3	3.7	1.7	2.6	4.0
RMSE (km)	20.3	21.0	22.3	20.3	20.8	22.5	20.5	21.1	22.6

**Table 11:** Simulation results of normal scenario with different σ.

	Proposed Algorithm			Relaxation Algorithm			Row Column Algorithm		
σ	$0.5^{\circ }$	$1^{\circ }$	$1.5^{\circ }$	$0.5^{\circ }$	$1^{\circ }$	$1.5^{\circ }$	$0.5^{\circ }$	$1^{\circ }$	$1.5^{\circ }$
Association accuracy	93%	68%	56%	94%	68%	55%	87%	65%	54%
Average run time (s)	2.4	0.7	0.3	9.2	9.8	9.0	7.3	7.7	7.1
Average number of false targets	1.2	2.2	2.4	1.0	2.2	2.4	1.3	2.3	2.5
RMSE (km)	11.3	32.4	60.9	11.0	32.3	60.9	11.5	32.9	61.9

**Table 12:** Simulation results of high clutter scenario with different σ.

	Proposed Algorithm			Relaxation Algorithm			Row Column Algorithm		
σ	$0.5^{\circ }$	$1^{\circ }$	$1.5^{\circ }$	$0.5^{\circ }$	$1^{\circ }$	$1.5^{\circ }$	$0.5^{\circ }$	$1^{\circ }$	$1.5^{\circ }$
Association accuracy	86%	59%	52%	85%	58%	51%	82%	55%	49%
Average run time (s)	2.8	0.8	0.4	40.2	41.3	38.6	28.5	28.6	26.4
Average number of false targets	3.0	4.5	4.8	3.1	4.5	4.9	3.3	4.7	5.2
RMSE (km)	12.2	33.2	62.7	12.2	33.3	62.9	12.8	33.4	63.5

**Table 13:** Simulation results of poor separated scenario with different σ.

	Proposed Algorithm			Relaxation Algorithm			Row Column Algorithm		
σ	$0.5^{\circ }$	$1^{\circ }$	$1.5^{\circ }$	$0.5^{\circ }$	$1^{\circ }$	$1.5^{\circ }$	$0.5^{\circ }$	$1^{\circ }$	$1.5^{\circ }$
Association accuracy	57%	42%	35%	57%	41%	35%	56%	41%	34%
Average run time (s)	2.3	0.6	0.3	9.8	9	9.2	7.5	7.1	7.3
Average number of false targets	1.5	1.4	1.4	1.5	1.5	1.4	1.7	1.6	1.6
RMSE (km)	20.3	30.7	41.4	20.3	30.9	41.6	20.5	31.2	41.8

**Table 14:** Simulation results of normal scenario with different $P_{D}$.

	Proposed Algorithm			Relaxation Algorithm			Row Column Algorithm		
$P_{D}$	0.9	0.7	0.5	0.9	0.7	0.5	0.9	0.7	0.5
Association accuracy	93%	70%	42%	94%	71%	41%	87%	57%	33%
Average run time (s)	2.4	2.2	2.0	9.2	4.5	2.1	7.3	4.1	1.9
Average number of false targets	1.2	1.8	2.2	1.0	1.8	2.2	1.3	2.3	2.6
RMSE (km)	11.3	12.8	15.5	11.0	12.7	15.5	11.5	12.8	15.8

**Table 15:** The true AOA measurements of the five targets measured by the five sensors.

	Target 1	Target 2	Target 3	Target 4	Target 5
Sensor 1	32.0${}^{\circ }$	$26.6^{\circ }$	$22.6^{\circ }$	32.3${}^{\circ }$	$29.7^{\circ }$
Sensor 2	$77.6^{\circ }$	69.9${}^{\circ }$	$62.9^{\circ }$	$72.9^{\circ }$	69.4${}^{\circ }$
Sensor 3	$97.6^{\circ }$	$90.0^{\circ }$	$82.4^{\circ }$	$91.8^{\circ }$	$88.2^{\circ }$
Sensor 4	$117.1^{\circ }$	110.1${}^{\circ }$	$102.4^{\circ }$	110.6${}^{\circ }$	$107.0^{\circ }$
Sensor 5	$157.4^{\circ }$	$153.4^{\circ }$	148.0${}^{\circ }$	$150.3^{\circ }$	147.7${}^{\circ }$

**Table 16:** Simulation results of a challenging scenario with different σ.

	Proposed Algorithm			Relaxation Algorithm			Row Column Algorithm		
σ	$0.5^{\circ }$	$1^{\circ }$	$1.5^{\circ }$	$0.5^{\circ }$	$1^{\circ }$	$1.5^{\circ }$	$0.5^{\circ }$	$1^{\circ }$	$1.5^{\circ }$
Association accuracy	69%	56%	49%	70%	55%	49%	67%	55%	48%
Average run time (s)	2.2	0.6	0.3	9.2	9.8	9.5	7.4	7.7	7.6
Average number of false targets	1.4	1.7	1.8	1.4	1.7	1.8	1.7	1.7	1.8
RMSE (km)	15.1	36.7	55.6	15.1	36.7	55.7	16.1	36.9	56.1

## Appendix A. CRLB of Localization Error

This appendix derives the CRLB of $x_{k}$, denoted as $\mathrm{CRLB}(x_{t})$. It is defined as the inverse of the Fisher matrix defined as [32]

(A1) $$F=-E\left[{\left(\frac{\partial \ln p\left(x_{k}\right)}{\partial x_{t}}\right)}^{T}\left(\frac{\partial \ln p\left(x_{k}\right)}{\partial x_{k}}\right)\right],$$

where $p\left(x_{k}\right)$ is the probability function

(A2) $$p\left(x_{k}\right)=\frac{1}{\sqrt{2\pi Q}}\exp\left\{-\frac{1}{2}{\left[z-h\left(x_{k}\right)\right]}^{T}Q^{-1}\left[z-h\left(x_{k}\right)\right]\right\}$$

where $z={\left[z_{1},z_{2},\ldots ,z_{S}\right]}^{T}$ is the measurement vector, $h\left(x_{t}\right)={\left[h\left(x_{k},x_{1}\right),h\left(x_{k},x_{2}\right),\ldots ,h\left(x_{k},x_{S}\right)\right]}^{T}$ and Q is the covariance matrix. Without loss of generality, it is assumed that the measurement noise of all sensors is independent and the covariance is equal to $\sigma^{2}$.

(A3) $$J=\frac{\partial \ln p\left(x_{k}\right)}{\partial x_{k}}=\frac{\partial h\left(x_{k}\right)}{\partial x_{k}}$$

After performing differentiation, the CRLB of $x_{k}$ is

(A4) $$CRLB\left(x_{k}\right)={\left(J^{T}Q^{-1}J\right)}^{-1}.$$
